# Multikinase Inhibitor Treatment in Thyroid Cancer

**DOI:** 10.3390/ijms21010010

**Published:** 2019-12-18

**Authors:** Ole Vincent Ancker, Marcus Krüger, Markus Wehland, Manfred Infanger, Daniela Grimm

**Affiliations:** 1 Department of Biomedicine, Aarhus University, Høegh-Guldbergsgade 10, 8000 Aarhus C, Denmark; ole@ancker.com; 2 Clinic for Plastic, Aesthetic and Hand Surgery, Otto von Guericke University, Leipziger Str. 44, 39120 Magdeburg, Germany; marcus.krueger@med.ovgu.de (M.K.); markus.wehland@med.ovgu.de (M.W.); manfred.infanger@med.ovgu.de (M.I.)

**Keywords:** thyroid cancer, multikinase inhibitors, lenvatinib, sorafenib, sunitinib, cabozantinib, pazopanib, vandetanib, adverse effects, clinical trials

## Abstract

Thyroid cancer is the most common endocrine malignancy. Most thyroid cancer types respond well to conventional treatment consisting of surgery and radioactive iodine (RAI) therapy. Unfortunately, some thyroid cancer types are resistant to surgical and RAI therapy. Multikinase inhibitors (MKIs) can be used in the treatment of advanced refractory thyroid cancers. The objective of this review is to give an update on MKI treatment (lenvatinib, sorafenib, sunitinib, cabozantinib, pazopanib, vandetanib) of thyroid cancer, regarding its efficacy and safety profile. We evaluated 212 articles through a PubMed search. A total of 20 articles met the inclusion and none the exclusion criteria. The studies showed promising progression-free survival rates compared to placebo treatment from earlier studies and similar or better results compared to the SELECT and DECISION trials. Adverse effects (AEs) are substantial in the treatment with MKIs. Almost all patients treated with these novel drugs experienced AEs. It is therefore crucial to focus on the management of AEs for a decent long-term outcome. The AEs are often more severe in patients with high efficacy of MKIs, which could indicate a correlation. Taken together, the novel therapeutic regimen with MKIs has shown favorable results in otherwise treatment-resistant thyroid cancer.

## 1. Introduction

Thyroid cancer is the most common endocrine cancer, affecting women in approximately 75% of cases [1]. Thyroid cancer accounts for about 550,000 cases per year worldwide. The global incidence rate is 10.2 per 100,000 among women, whereas it is 3.1 per 100,000 for men. Thyroid cancer is frequent in both men and women in the Republic of Korea and is the most frequent cancer among Korean women. [2]. In the last 30 years, an increase in new cases of thyroid cancer has been seen almost all over the world. Even though the incidence rate of thyroid cancer is increasing, the mortality rate is more stable [3]. The steady mortality rate may be due to the current treatment possibilities available for thyroid cancer. In Denmark, the treatment of thyroid cancer is a highly specialized task. Surgery is, for most thyroid cancer cases, the first choice, often together with radioactive iodine (RAI) as an adjuvant treatment [4].

In general, treatment of differentiated thyroid cancer shows promising results, with a long-term survival rate near 90%. Unfortunately, the poorly differentiated thyroid cancer types show a more discouraging long-term survival rate of close to 10%. The discouraging long-term survival for poorly differentiated thyroid cancers (PDTCs) is a result of their resistance to the standard treatment options. During recent years, a new treatment option with multikinase inhibitors (MKIs) such as sunitinib, sorafenib, lenvatinib, pazopanib, vandetanib, and cabozantinib has shown promising results in otherwise treatment-refractory thyroid cancer [5,6].

The objective of this work is to give an update on MKI treatment of thyroid cancer by describing the differences in thyroid cancer and the mechanisms involved in the current treatment, and by providing a systematic overview of studies published in 2018 and 2019 investigating the effects of MKI treatment in thyroid cancer. The hypothesis of this review is that the MKI treatment of advanced and RAI-refractory thyroid cancer shows beneficial effects on these cancer types and is developing both in terms of the efficacy and the safety profiles.

## 2. Thyroid Cancer

Thyroid cancer is often classified into the following groups: papillary thyroid cancer (PTC), follicular thyroid cancer (FTC), Hürthle cell carcinoma (HCC), medullary thyroid cancer (MTC), and anaplastic thyroid cancer (ATC). These subgroups vary in aggressivity and thereby in prognosis [7]. The differentiated thyroid cancers (DTCs), which include PTC, FTC, and HCC, are the most common types. The DTCs derive from follicular thyroid cells. They have a less aggressive nature and thus the best prognosis. The poorly differentiated thyroid carcinomas (PDTCs) show a lack of differentiation and a higher tendency to metastasize. This makes their treatment more challenging, which ultimately results in a much worse prognosis [8,9].

The survival rates for thyroid cancer depend on the cancer type and vary greatly. In Denmark, most patients suffering from PTC, which accounts for 65% of primary thyroid cancers, have a five-year survival of 91%. The incidences of FTC (20%), MTC, or ATC (both 7%) are lower. Whereas FTC (five-year survival: 80%) and MTC (five-year survival: 70%) show higher survival rates, the diagnosed ATC has the poorest five-year survival of only 12% [4]. It has been suggested that the 5- and 10-year survival rates of PDTCs are 50% and 25%–35%, respectively, and that 4%–7% of all thyroid cancers are PDTCs [10].

These numbers show that the overall survival (OS) of thyroid cancer is relatively high, primarily due to surgery and RAI. Local recurrence of the treated thyroid cancer occurs in up to 20% of cases. Unfortunately, the recurrent state of the cancer is poorly differentiated, which makes conventional treatment troublesome [11].

The conventional thyroid cancer therapy consists of surgery, RAI, and suppression of thyroid-stimulating hormone (TSH). The therapy shows decent results compared to treatment of several other cancer types [2,12]. The surgical aim is the resection of the tumor in its whole. The surgical procedure often includes a total thyroidectomy and a neck dissection. RAI treatment is often used as an adjuvant treatment after surgery. RAI treatment uses the ability of the thyroid cells to absorb iodine. The iodine in RAI treatment is radioactive and destroys the cells it enters [12]. TSH suppression is used after surgery and RAI treatment because the DTCs express the TSH receptors and use the TSH to grow [13]. The novel treatment with MKIs has shown favorable results in otherwise treatment-resistant thyroid cancer [14].

## 3. Multikinase Inhibitors

The majority of thyroid cancer types are well differentiated, which contributes to the optimistic prospects when treating thyroid cancer. The advanced DTCs, PDTCs, and ATCs, on the other hand, must be targeted differently to improve the prognosis.

Angiogenesis is the formation of new vessels from pre-existing ones. It plays a vital role in embryogenesis and becomes less critical in healthy adults. Tumors can grow up to 1–2 mm without vascularity of its own before demanding a bigger nutrient and oxygen supply than diffusion can bring. Angiogenesis is a normal physiological process, but it becomes pathophysiological when the tumor uses it developmentally [15,16].

The vascular endothelial growth factor (VEGF) is a crucial contributor in angiogenesis. VEGF consists of VEGF-A, VEGF-B, VEGF-C, VEGF-D, and the placental growth factor (PGF). These VEGF ligands bind to different tyrosine kinase receptors, the VEGF receptors (VEGFR), comprising VEGFR-1, VEGFR-2, and VEGFR-3.

The induction of VEGFR-1 and -2 activates angiogenesis, whereas the induction of VEGFR-3 activates embryogenic angiogenesis and lymphangiogenesis. VEGFR-1 has VEGF-A, VEGF-B, and PGF as its ligands, VEGFR-2 has VEGF-A and proteolytically modified VEGF-C and -D. Finally, VEGFR-3 is activated by VEGF-C and -D [17,18]. Rearranged during transfection (RET) and fibroblast growth factor receptor (FGFR) are also tyrosine kinase receptors of high importance in the development of thyroid cancer. The tyrosine kinase receptors are located in the cell membrane. Ligands bind to their corresponding receptors, activating an intracellular phosphorylation cascade, ultimately resulting in angiogenesis and tumor growth [11].

**Figure 1:**
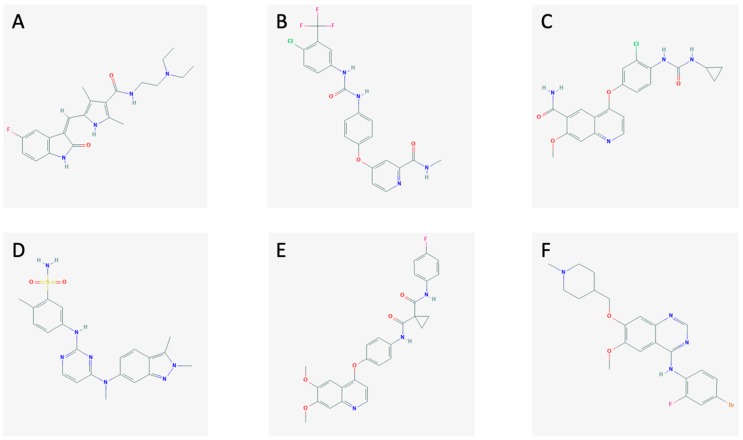
The chemical structures of (**A**) sunitinib, (**B**) sorafenib, (**C**) lenvatinib, (**D**) pazopanib, (**E**) cabozantinib, and (**F**) vandetanib.

The MKIs (sunitinib, sorafenib, lenvatinib, pazopanib, cabozantinib, and vandetanib; Figure 1) block the activation of tyrosine kinases in different ways.

Sunitinib (Figure 1A) is an MKI currently approved for the treatment of gastrointestinal stromal tumors, renal carcinoma, and pancreatic neuroendocrine tumors. It blocks the signals from VEGFR-1, -2, -3, the platelet-derived growth factor receptor (PDGFR), the stem cell factor receptor (c-KIT), FMS-like tyrosine kinase 3 (FLT-3), and RET [19,20,21].

Sorafenib (Figure 1B) is an MKI which is approved to treat advanced renal cell carcinoma, unresectable hepatocellular carcinoma, and metastatic differentiated thyroid cancer. It targets VEGFR-2 and -3, FLT-3, PDGFRβ, c-KIT, RET, and RAF [21,22].

Lenvatinib (Figure 1C) is a new MKI approved by the U.S. Food and Drug Administration (FDA) and European Medicines Agency (EMA) for the therapy of RAI-refractory differentiated thyroid cancer, advanced renal cell carcinoma, and, just recently, for unresectable hepatocellular carcinoma [23]. It inhibits the pathways through VEGFR-1, -2, -3, FGFR-1, -2, -3, -4, PDGFRα, RET, and c-KIT [24,25].

Pazopanib (Figure 1D) is an MKI approved to treat advanced renal cell carcinoma and advanced soft tissue carcinoma. Furthermore, it is currently being tested as a treatment for MTC showing promising results [26]. It hits VEGFR-1, -2, -3, PDGFR, and c-KIT [27].

Cabozantinib (Figure 1E) is an MKI targeting the VEGFR-2, RET, and the hepatocyte growth factor receptor (MET). It is used in metastatic renal cell carcinoma [26,28].

**Figure 2:**
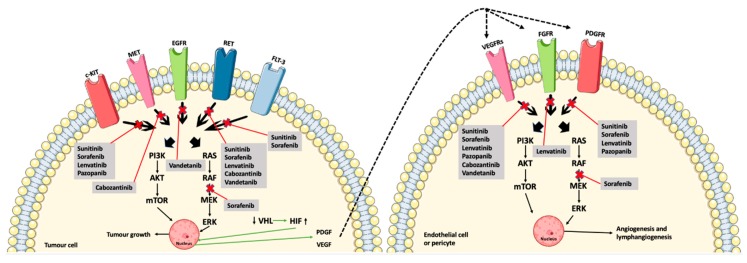
The MKIs (sunitinib, sorafenib, lenvatinib, pazopanib, cabozantinib, and vandetanib) block signaling from the tyrosine kinase receptors, thus preventing phosphorylation and, ultimately, angiogenesis and tumor growth. Furthermore, the interplay between the tumor cells, endothelial cells, and pericytes are shown by the downregulation of the tumor suppressor, VHL, and thereby less inhibition of HIF, which causes an increased induction of angiogenesis due to the production of VEGF and PDGF (green arrows) [11,24,26,29]. Abbreviations: RAS (rat sarcoma protein), RAF (rapidly accelerated fibrosarcoma kinase), MEK (mitogen-activated protein kinase kinase), ERK (mitogen-activated protein kinase), PI3K (phosphoinositide 3-kinase), AKT (protein kinase B), mTOR (mammalian target of rapamycin), VHL (von Hippel–Lindau tumor suppressor), HIF (hypoxia-inducible factor). Crossed-out arrows represent the inhibited signaling pathways by the indicated drugs.

Vandetanib (Figure 1F) is an MKI approved for advanced MTC. It obstructs signaling from VEGFR-2, RET, and the epidermal growth factor receptor (EGFR) [26]. The mechanisms of action of the MKIs are demonstrated in Figure 2, along with the interplay between the tumor cells and the endothelial cells and pericytes.

## 4. Methods

The literature search was performed by example of the Preferred Reporting Items for Systematic Reviews and Meta-Analyses (PRISMA) guidelines [30] and by using the PubMed database.

### 4.1. Eligibility Criteria

Studies investigating the efficacy or safety of MKI treatment of thyroid cancer in patients aged 18 years or older were included in this review. The exclusion criteria comprised languages other than English, studies on cell cultures or animals, and articles published before 01.01.2018. Systematic reviews, meta-analyses, and case reports were not included.

### 4.2. Information Sources

The literature search for this review was conducted on 3 November 2019 by searching the database PubMed.

### 4.3. Search

The search was done with the following terms: (“Thyroid Neoplasms”[Mesh] OR “Thyroid Carcinoma, Anaplastic”[Mesh] OR “Thyroid Cancer, Papillary”[Mesh] OR “Thyroid cancer, medullary” [Supplementary Concept] OR “Thyroid cancer, follicular” [Supplementary Concept] OR “thyroid cancer”[title/abstract]) AND (“Protein Kinase Inhibitors”[Mesh] OR “multikinase inhibitor” OR “tyrosine kinase inhibitor” OR “lenvatinib” OR “sunitinib” OR “sorafenib” OR “vandetanib” OR “pazopanib” OR “cabozantinib”).

### 4.4. Study Selection

**Figure 3:**
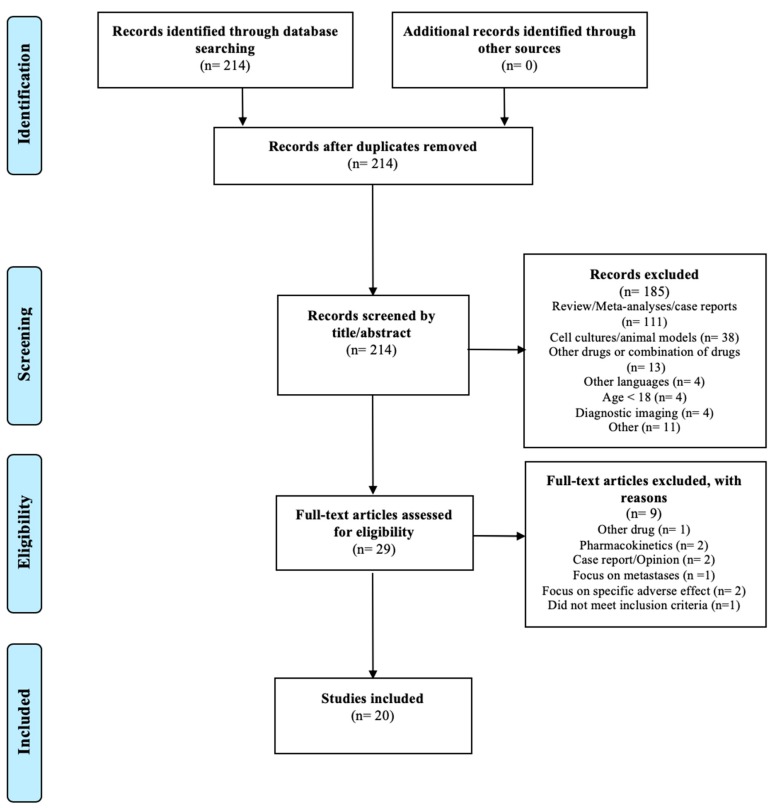
PRISMA flow diagram modified from Liberati et al. [30].

The search resulted in 214 articles. A total of 185 articles were excluded by screening the title and the abstract for the eligibility criteria; 20 of the remaining 29 articles met the inclusion criteria and none of the exclusion criteria. The study selection is illustrated in the PRISMA flow diagram shown in Figure 3.

### 4.5. Data Collection Process

The authors of the review completed the search. Data were included if fulfilling the inclusion criteria and not fulfilling any of the exclusion criteria.

### 4.6. Data Items

The criterium “thyroid cancer” covers different types of cancer. The histological difference is of great importance when treating cancer. Therefore, the type of cancer had to be verified. The criterium “efficacy or safety of multikinase inhibitor” includes the direct effect on the disease (e.g., by measuring progression-free survival (PFS)) but also the adverse effects (AEs) detected when treating with MKIs.

The information obtained from the articles featured study type, type of thyroid cancer, name and starting dose of MKI, characteristics of study population (number of participants, gender, age, country), effect on the thyroid cancer disease, and AEs. To make the results of the studies comparable, it was decided to use PFS as a result of efficacy if possible. In studies where PFS was not disclosed, the response rate (RR) was used. RR is defined as complete response or partial response. The standardized Response Evaluation Criteria in Solid Tumors (RECIST) [31] must be used for the evaluation of PFS and RR.

Only data where MKIs were used as first-line monotherapy were included in the results if possible. Locati et al. [32] did not mention the method of evaluating the efficacy of lenvatinib, but since the study was made by example of the SELECT trial [24], it is assumed that the evaluation was based on RECIST. In Iwasaki et al. [33], there was an incongruence regarding the sex of the participants: the text talked about a trial population of 15 women (65.2%) and 8 men, but the table in the article registered 16 women (69.6%) and 7 men. It was assumed that the correct proportion was given in the table. Information obtained from the studies is summarized in Table 1 and Table 2.

### 4.7. Risk of Bias in Individual Studies

In Molina-Vega et al. [34], the PFS was measured on first-, second-, and third-line treatment, which increased the risk of bias. The patients switching MKI because of AEs may have been more sensitive to the anticancer drug and showed a better efficacy even though they experienced a lower tolerability. The exact opposite might also be the case. In both instances, there is a risk that the result is biased.

## 5. Results

The results of the 20 included studies are presented in Table 1 and Table 2. The studies mainly focus on lenvatinib, but sorafenib and vandetanib are also encompassed. An overview of the studies and characteristics of the patients as well as the efficacy is provided in Table 1. The total prevalence of AEs and the events observed in more than 50% of the patients are presented in Table 2.

For RAI-refractory DTC patients treated with lenvatinib, the PFS varies a lot from the lowest PFS found, 7.2 months [35], to the highest value, 33.1 months [36].

Patients with RAI-refractory DTC treated with sorafenib show some variability as well. The lowest PFS for sorafenib-treated patients was 9.7 months, published in Kim et al. [40], whereas the highest value of 18 months was published in Molina-Vega et al. [34].

Hu et al. [37] tested two doses of vandetanib (150 mg and 300 mg) in patients with advanced MTC. They found RR values for 150 mg of 20% and for 300 mg of 29.3%.

Three studies investigated the effect of lenvatinib in patients with ATC: Takahashi et al. [49] measured a PFS of 7.4 months. Koyama et al. [44] reported an OS of 165 days and an RR of 60%. Moreover, Iwasaki et al. [33] published an OS of 166 days and an RR of 17.4%.

Kocsis et al. [43] treated patients suffering from advanced MTC with sorafenib. They found a PFS of 19.1 months. Takahashi et al. [49] found a PFS of 9.2 months in patients with advanced MTC treated with lenvatinib.

Table 2 shows that AEs are almost ubiquitous. Locati et al. [32] found the lowest prevalence of AEs, with a value of 87.2%, whereas the other studies found a prevalence close to or of 100%. A pattern of the most frequently appearing AEs can be observed in Table 2. Fatigue, palmar-plantar erythrodysesthesia syndrome, diarrhea, hypertension, and proteinuria are present in at least 50% of the patients with AEs in most of the studies.

## 6. Discussion

MKI treatment of RAI-refractory cancer has shown promising results. Unfortunately, it does not remove all issues relating to RAI-refractory cancer. Almost all studies of MKIs show a significant quantity and grade of AEs. Furthermore, not all RAI-refractory thyroid cancer patients show an equally good response [24]. The studies evaluated in this review showed some differences in their outcome, but indeed also resemblances.

Most of the studies given in this review were retrospective, observational studies. All of the studies were conducted after the publication of the SELECT [24] and DECISION trials [52]. Most of the investigations attempted to compare the promising results previously found with patients treated with MKIs with their own division or region. Retrospective, observational studies cannot entirely avoid selection bias, and it is therefore essential to keep this in mind when comparing them with double-blinded, randomized clinical trials. The other studies in this review were prospective, observational studies, post hoc analyses based on data from the SELECT trial [24], one nonrandomized phase II study, and one double-blinded randomized study. The post hoc analyses were based on data from patients from a randomized, double-blinded study, but these studies were, as the retrospective studies, affected by selection bias as they chose to focus on only some of the data.

Additionally, most of the included studies had very few participants, ranging from 5 to 190 patients; this lowers the meaningfulness of the studies. A strength of the studies is that they mostly represented the picture of an everyday clinical use of MKIs. Because of the mentioned possible bias, it is important to look critically at some of the results presented.

The highest PFS in lenvatinib-treated patients was 33.1 months (95% CI, 27.8–44.6) in Gianoukakis et al. [36], which is up to 3 times as high as published in some of the other included studies [32,35]. It is worth noting that Gianoukakis et al. [36] was a post hoc analysis focusing only on patients with a response in the SELECT trial [24], thus sorting out the patients with early progression of disease and therefore heightening the PFS compared to other studies.

It has been suggested that men present with more aggressive and advanced thyroid cancers [53]. The sex may have an impact on the outcome of the MKI treatment and, as mentioned earlier, women are affected by thyroid cancer in three out of four cases [1]. Interestingly, the female participants varied from 0% to 83% in the included studies, which could affect the results.

Age is a central factor in thyroid cancer [54]. Therefore, it is necessary to consider this aspect when studying the results of the articles. Most of the studies included had a large age span. Takahashi et al. [49] had, for example, an age span from 21 to 84 years. This broad representation of age might influence the results.

Moreover, earlier treatment with an MKI is a possible confounder. Patients previously treated with an MKI may have different responses than naïve patients and thereby this may impact the results. Kocsis et al. [43] ruled out all patients who had been treated with another systematic anticancer treatment, whereas, for example, 62% of the patients included in Balmelli et al. [35] had received sorafenib treatment before enrolment.

Locati et al. [32] reported that their patients had a worse performance status compared to the SELECT trial [24]. The condition of the patients is crucial when evaluating the efficacy of the treatment but certainly, also the tolerability. Patients with a lower resistance to AEs or patients who are more likeable to interrupt or reduce the treatment will contribute to reducing the efficacy of the drug over time.

The different types of cancers like RAI-refractory DTC, MTC, and ATC show a diversity in outcomes. Not surprisingly, ATC shows the worst survival rates, with an OS of 165 days [44] and 166 days [33] and a PFS of 7.4 months [49]. These results support the current knowledge regarding ATC having a much worse prognosis compared to the other types of thyroid cancer, even when treated with MKIs.

MTC showed a better outcome, with a PFS of 9.2 months [49] and 19.1 months [43] and an RR in 20% and 29.3% [37]. Direct comparison of the results is problematic as each study investigated a different drug. However, it can be suggested from the results that MTC shows more promising treatment outcomes than ATC when treated with MKIs and that a higher dose of vandetanib possibly raises the RR.

DTC varied from a PFS of 7.2 to 33.1 months. Generally, DTCs showed higher survival rates than ATC and MTC, even though the range was extensive. Patients treated with sorafenib had a PFS in the range of 9.7 months to 18 months, whereas lenvatinib varied from 7.2 months to 33.1 months. The PFS could suggest the higher treatment potential of lenvatinib compared with sorafenib, but it is vital to keep the previously mentioned reservations in mind when assessing the results. The SELECT trial [24] found a PFS in lenvatinib-treated patients of 18.3 months (95% CI, 15.1–N/R) and in placebo-treated patients 3.6 months (95% CI, 2.2–3.7). The DECISION trial [52], a phase III study comparing sorafenib to a placebo, demonstrated a median PFS for sorafenib-treated patients of 10.8 months and, for the placebo-treated patients, 5.8 months. The comparison indicates that the included studies have shown similar or even better results.

One study [47] tried to determine the predictive and prognostic factors of MKI treatment. The authors concluded that high tumor burden and tumor-related symptoms were independent prognostic factors. Therefore, the authors suggested that MKI treatment should be started before the tumor burden is too immense and before tumor-related symptoms are dominating.

The AEs of MKI are, apart from the efficacy, a pivotal point to address. The studies included substantiated the importance of focusing on AEs given the prevalence of AEs ranging from 87.2% to 100%. It is well known that MKIs cause AEs, with proteinuria, diarrhea, hypertension, and palmar-plantar erythrodysesthesia syndrome being some of the most common [55,56]. The AEs play a substantial part in the treatment with MKIs. Dose interruption and reduction harm the long-term efficacy of the drugs [57]. Several of the included studies [35,36,37,40] underlined that early management of the AEs is crucial for the prognosis.

In the included studies, a minimum of 50% of all the sorafenib-treated patients experiencing AEs acquired palmar-plantar erythrodysesthesia. In the patients treated with lenvatinib, events of palmar-plantar erythrodysesthesia were not observed to the same extent. It was, instead, hypertension that seemed to be the dominating AE. Kim et al. [41] found that patients with palmar-plantar erythrodysesthesia showed a better PFS. The authors suggested that the higher PFS in these patients might be caused by a more effective blockade of the involved receptors, thereby causing both an improved response but also stronger AEs. It has been suggested that hypertension could be a potential biomarker for MKI anticancer treatment [21]. Wirth et al. [50] contributes to the hypothesis that hypertension could be a biomarker for effective MKI treatment since they found hypertension to be significantly correlated with clinical outcomes compared to the SELECT trial [24].

## 7. Conclusions

In conclusion, MKIs still show promising results in the treatment of advanced and RAI-refractory thyroid cancer. Applying the PRISMA guidelines, we found that only studies investigating lenvatinib, sorafenib, and vandetanib were eligible based on the inclusion and exclusion criteria. The survival rates are all encouraging compared to placebo from earlier studies. The results of the included studies show a similar or superior PFS than the two widely acknowledged studies: the SELECT [24] and the DECISION trial [52]. However, it is important to keep the study designs of the included studies in mind. The AEs are unfortunately widespread and often very serious in MKI-treated patients. The management of the AEs is, therefore, essential for the long-term efficacy of MKIs.

## 8. Outlook

This review only included studies concerning the monotherapy and first-line treatment with MKIs. The results could potentially be even more optimistic if looking at MKIs also used as second- or third-line therapy. In the future, it might be of great value to study the effect and safety of MKIs in combination with, for example, immunotherapy or other anticancer drugs. Moreover, it could be interesting to investigate possible biomarkers for a satisfactory response to MKI treatment further (e.g., the possible correlation between AEs and efficacy).

## Figures and Tables

**Table 1:** Overview of the literature regarding the efficacy of MKI treatment.

Reference	Type of Study	Drug + Starting Dose	Objective	Type of Cancer	Patient Characteristics	Efficacy Outcome	Country
Balmelli et al., 2018 [35]	Retrospective	Lenvatinib, 24 mg	Efficacy and toxicity	RAI-refractory, metastatic DTC	Patients: 13 Median age: 72 (range: 37–81) Female: N/A	PFS: 7.2 months (95% CI, 0.8–13.7)	Switzerland
Gianoukakis et al., 2018 [36]	Randomized, double-blind, post hoc analysis	Lenvatinib, 24 mg	Duration of survival in responders	RAI-refractory DTC. PTC, FTC, HCC, and PDTC	Patients: 157 Female: 53.5% Age: ≤65: 66.2% Age >65: 33.8%	PFS: 33.1 months (95% CI, 27.8–44.6)	America, Europe, Asia, and Australia
Hu et al., 2019 [37]	Randomized, double-blind	Vandetanib, 150 mg, 300 mg	Efficacy and tolerability	Unresectable, locally advanced or metastatic MTC	Patients: 81 Female: 33.3% Mean age: 52.5	150 mg: RR: 20% (95% CI, 10.5%–34.8%) 300mg: RR: 29.3% (95% CI, 17.6%–44.5%)	Nine countries
Iwasaki et al., 2018 [33]	Retrospective	Lenvatinib, 24 mg, 20 mg, 14 mg, 10 mg	Safety and efficacy	ATC	Patients: 23 Female: 69.6% Median age: 77 (range: 42–89)	RR: 17.4% OS: 166 days	Japan
Iwasaki et al., 2019 [38]	Retrospective	Sorafenib, lenvatinib. Dose unknown	Efficacy	Metastatic PTC and FTC	Patients: 56 Female: 62.5% Median age: 70 (range 41–84)	RR: 28.5%	Japan
Jerkovich et al., 2019 [39]	Retrospective	Sorafenib	Efficacy and safety	PTC, FTC, and HCC	Patients: 18 Female: 54.6% ^a^ Median age: 61 (range 36–75)	Median PFS: 16.5 months	Argentina
Kim et al., 2018 [40]	Retrospective	Sorafenib Mean daily dose 666 ± 114 mg	Efficacy and safety	RAI-refractory DTC; PTC, FTC, HCC, and PDTC	Patients: 98 Female: 69% Median age: 65.6 (range 57.7–72.2)	PFS: 9.7 months (range 4.5–16.7)	Korea
Kim et al., 2019 [41]	Retrospective	Sorafenib Mean daily dose: 602 mg	Efficacy and safety	RAI-refractory locally advanced or metastatic DTC; PTC, FTC, and PDTC	Patients: 85 Female: 61% Median age: 55 (range 22–81)	Median PFS: 14.4 months (range 1.6–92.2)	Korea
Kim et al., 2019 [42]	Retrospective	Sorafenib ≤400 mg–800 mg Lenvatinib 20 mg	Safety	RAI-refractory locally advanced or metastatic DTC; PTC, FTC, HCC, and PDTC.	Patients Lenvatinib: 23 Female: 60.9% Median age: 59.7 (range 38.9–74.4) Patients Sorafenib: 48 Female: 58.3% Median age: 62.0 (range 32.6–79.0)	Not available (N/A)	Korea
Kocsis et al., 2018 [43]	Prospective	Sorafenib 400 mg × 2	Efficacy and safety	Metastatic, progressive, or symptomatic MTC	Patients: 10 Female: 60% Mean age: 51.7 (range 25–71)	Median PFS: 19.1 months	Hungary
Koyama et al., 2018 [44]	Retrospective	Lenvatinib 24 mg	Efficacy and safety	ATC	Patients: 5 Female: 0% Mean age: 58.8	Median OS: 165 days. RR: 60%	Japan
Locati et al., 2019 [32]	Retrospective	Lenvatinib 24 mg for 71% of patients	Efficacy and toxicity	RAI-refractory DTC	Patients: 94 Female: 48.9% Median age: 60 (range 23–82)	PFS: 10.8 months (95% CI, 7.7–12.6)	Italy
Molina-Vega et al., 2018 [34]	Retrospective	Sorafenib: 800 mg or 400 mg Lenvatinib: mean dose 21,6 mg	Efficacy and safety	RAI-refractory metastatic DTC; PTC, FTC, and HCC.	Patients Sorafenib: 16 Patients Lenvatinib: 1 Female: 47.1% Mean age: 64.7	Median PFS: 18 months	Spain
Nervo et al., 2018 [45]	Retrospective	Lenvatinib 24 mg	Efficacy and safety	RAI-refractory DTC; PDTC, PTC, and FTC	Patients: 12 Female: 75% Median age: 61 (range 51.5–68)	PFS 6m: 63.6% (95% CI, 29.7–84.5)PFS 12m: 54.6% (95% CI, 22.9–78.0)	Italy
Sugino et al., 2018 [46]	Retrospective	Lenvatinib 24 mg	Efficacy	RAI-refractory DTC; PTC and FTC	Patients: 29 Female: 69% Median age: 66 (32–81)	Median PFS: 24.3 months	Japan
Suzuiki et al., 2019 [47]	Retrospective	Lenvatinib 24 mg	Prognostic and predictive factors	RAI-refractory DTC; PTC, FTC, PDTC	Patients: 26 Female: 69.2% Median age: 64 (range 30–83)	Two-year PFS: 58.4%	Japan
Tahara et al., 2019 [48]	Randomized double-blind, post hoc analysis	Lenvatinib 24 mg	Efficacy in dose-interrupted patients	Progressive, RAI-refractory PTC, PDTC, FTC, and HCC	Patients group 1^b^:134 Female: 49.3% Median age: 61.5 (range 27–83) Patients group 2^c^: 127 Female: 55.1% Median age: 65.0 (range 39–89)	Group 1:PFS: not reached (N/R)Group 2:PFS: 12.8 months (95% CI, 9.3–16.5)	America, Europe, Asia, and Australia
Takahashi et al., 2019 [49]	Nonrandomized phase II study	Lenvatinib 24 mg	Safety and efficacy	RAI-refractory DTC, MTC, and ATC.	Patients: 51 Female: 59% Median age: 61 (21–84)	PFS: RAI-Refractory DTC: 25.8 months (95% CI, 18.4–N/R)MTC: 9.2 months (95% CI, 1.8–N/R) ATC: 7.4 months (95% CI, 1.7–12.9)	Japan
Wirth et al., 2018 [50]	Randomized double-blind, post hoc analysis	Lenvatinib 24 mg	Efficacy and safety in patients with treatment-emergent hypertension	Progressive, RAI-refractory PTC, PDTC, FTC, and HCC^d^.	Patients: 190 Female: N/A Age: N/A	Median PFS: 18.8 months (95% CI, 16.5–N/R)	America, Europe, Asia, and Australia
Yamazaki et al., 2019 [51]	Retrospective	Lenvatinib 24, 20, 14, 10 mg	Compare low dose lenvatinib to full dose, 24 mg	DTC; PTC and FTC	Full dose: Patients: 30 Female: 67% Median age: 68 (range 47–83) Low dose: Patients: 6 Female: 83% Median age: 77 (range 41–84)	Full doseMedian PFS: 696 days (95% CI, 318–N/R) Low dose: Median PFS: N/R (95% CI, 124 days–N/R)	Japan

^a^ Based on 22 patients. Only 18 participated in the study; ^b^ Group 1: duration of dose interruption <10% of total treatment time; ^c^ Group 2: duration of dose interruption ≥10% of total treatment time; ^d^ In the SELECT trial. Unclear in these selected patients. N/A: not available, N/R: not reached

**Table 2:** Overview of adverse effects (AEs).

Reference	Drug + Starting Dose	Prevalence of AEs	AEs in ≥50% of Patients
Balmelli et al., 2018 [35]	Lenvatinib, 24 mg	92%	Fatigue (50%)
Gianoukakis et al., 2018 [36]	Lenvatinib, 24 mg	80.8%^a^	N/A
Hu et al., 2019 [37]	Vandetanib, 150 mg, 300 mg	150 mg: 97.5% 300 mg: 97.6%	None ≥50%
Iwasaki et al., 2018 [33]	Lenvatinib, 24 mg, 20 mg	100%	Hypertension (91%) Fatigue and anorexia (65%) Proteinuria (61%)
Iwasaki et al., 2019 [38]	Sorafenib, lenvatinib	N/A	N/A
Jerkovich et al., 2019 [39]	Sorafenib	90%	Palmar-plantar erythrodysesthesia syndrome (67%) Diarrhea (52%) Hypertension (52%)
Kim et al., 2018 [40]	Sorafenib Mean daily dose 666 ± 114 mg	95%	Palmar-plantar erythrodysesthesia syndrome (76%)
Kim et al., 2019 [41]	Sorafenib Mean daily dose: 602 mg	64%^b^	N/A
Kim et al., 2019 [42]	Sorafenib: ≤400 mg–800 mg Lenvatinib: 20 mg	N/A	Lenvatinib: Palmar-plantar erythrodysesthesia syndrome (56.5%) Diarrhea (82.6%) Hypertension (78.3%) Decreased weight (52.2%) Sorafenib: Palmar-plantar erythrodysesthesia syndrome (87.5%) Diarrhea (62.5%) Anorexia (60.4%) Alopecia (56.3%) Mucositis (52.1%) Generalized weakness (50%)
Kocsis et al., 2018 [43]	Sorafenib 400 mg × 2	100%	Fatigue (60%) Palmar-plantar erythrodysesthesia syndrome (50%) Rash/dermatitis (50%)
Koyama et al., 2018 [44]	Lenvatinib 24 mg	100%	Proteinuria (100%) Hypothyroidism (80%) Hypertension (80%) Fatigue (80%) Anorexia (80%) Decreased weight (80%)
Locati et al., 2019 [32]	Lenvatinib 24 mg for 71% of patients	87.2%	N/A
Molina-Vega et al., 2018 [34]	Sorafenib: 800 mg or 400 mg Lenvatinib: mean dose 21.6 mg	100%	Sorafenib: Fatigue (68.7%) Palmar-plantar erythrodysesthesia syndrome (68.7%) Diarrhea (62.5%) Lenvatinib: Fatigue (100%) Hypertension (80%) Palmar-plantar erythrodysesthesia syndrome (60%) Diarrhea (60%)
Nervo et al., 2018 [45]	Lenvatinib 24 mg	100%	Decreased weight (91.7%) Palmar-plantar erythrodysesthesia syndrome (91.7%) Hypertension (75%) Nausea (75%) Diarrhea (66.7%) Fatigue (58.3%) Oral mucositis (58.3%) Decreased appetite (58.3%) Myalgia (58.3%) Arthralgia (50%)
Sugino et al., 2018 [46]	Lenvatinib 24 mg	100%	Hypertension (75.9%) Palmar-plantar erythrodysesthesia syndrome (58.6%)
Suzuki et al., 2019 [47]	Lenvatinib 24 mg	96.2%^d^	Proteinuria (61.5%)^d^ Malaise (57.7%)d
Tahara et al., 2019 [48]	Lenvatinib 24 mg	Group 1^e^: 100% Group 2^f^: 99.2%	Group 1: Diarrhea (73.9%) Hypertension (69.4%) Decreased weight (56.7%) Group 2: Hypertension (69.3%) Decreased appetite (62.2%) Diarrhea (58.3%)
Takahashi et al., 2019 [49]	Lenvatinib 24 mg	100%	RAI-refractory DTC: Hypertension (96%) Palmar-plantar erythrodysesthesia syndrome (92%) Fatigue (80%) Decreased appetite (68%) Stomatitis (68%) Proteinuria (60%) Diarrhea (60%) Arthralgia (56%) MTC: Decreased appetite (100%) Hypertension (89%) Palmar-plantar erythrodysesthesia syndrome (89%) Diarrhea (89%) Fatigue (78%) Proteinuria (67%) Insomnia (56%) ATC: Hypertension (82%) Decreased appetite (82%) Fatigue (59%) Proteinuria (59%) Nausea (59%) Insomnia (56%)
Wirth et al., 2018 [50]	Lenvatinib 24 mg	100%^g^	Hypertension (100%)^g^
Yamazaki et al., 2019 [51]	Lenvatinib 24, 20, 14, 10 mg	Full dose: Unknown Low dose: 100%	Full dose: Hypertension (93%) Proteinuria (77%) Palmar-plantar erythrodysesthesia syndrome (77%) Low dose: Hypertension (100%) Proteinuria (83%) Palmar-plantar erythrodysesthesia syndrome (67%)

^a^ ≥Grade 3 AE; ^b^ marked as severe; ^c^ also includes lenvatinib as second-line treatment; ^d^ dose reduction because of AEs; ^e^ Group 1: duration of dose interruption <10%; ^f^ Group 2: duration of dose interruption ≥10%; ^g^ treatment-emergent hypertension was an inclusion criterium. N/A: not available

## Abbreviations

AE(s)	Adverse effect(s)
AKT	Protein kinase B
ATC	Anaplastic thyroid cancer
c-KIT	Stem cell factor receptor
DTC	Differentiated thyroid cancer
EGFR	Epidermal growth factor receptor
ERK	Mitogen-activated protein kinase
FGFR	Fibroblast growth factor receptor
FLT-3	FMS-like tyrosine kinase 3
FTC	Follicular thyroid cancer
HCC	Hürthle cell carcinoma
HIF	Hypoxia-inducible factor
MEK	Mitogen-activated protein kinase kinase
MET	Hepatocyte growth factor receptor
MKI(s)	Multikinase inhibitor(s)
MTC	Medullary thyroid cancer
mTOR	Mammalian target of rapamycin
N/A	Not available
N/R	Not reached
OS	Overall survival
PDGFR	Platelet-derived growth factor receptor
PDTC(s)	Poorly differentiated thyroid cancer(s)
PFS	Progression-free survival
PGF	Placental growth factor
PI3K	phosphoinositide 3-kinase
PRISMA	Preferred Reporting Items for Systematic Reviews and Meta-Analyses
PTC	Papillary thyroid cancer
RAF	Rapidly accelerated fibrosarcoma kinase
RAI	Radioactive iodine
RAS	Rat sarcoma protein
RET	Rearranged during transfection
RECIST	Response Evaluation Criteria in Solid Tumors
RR	Response rate
TSH	Thyroid-stimulating hormone
VEGF	Vascular endothelial growth factor
VEGFR	Vascular endothelial growth factor receptor
VHL	Von Hippel–Lindau tumor suppressor
